# Three Provincial Hospitals

**Published:** 1894-02-24

**Authors:** 


### THE INSTITUTIONAL WORKSHOP.

### WITHIN THE HOSPITALS.

### THREE PROVINCIAL HOSPITALS.

(BY A VAGRANT CORRESPONDENT.)

### ALNWICK INFIRMARY.

About the distance of a bowshot from the historic old border fortress and castle of Alnwick stands the infirmary. In striking contrast are the luxury and splendour of the seat of the Duke of Northumberland to the mean accommodation for the sick poor in Alnwick town. An old house in a row of old houses, only distinguishable by its name on a black board across its entrance, is the Hotel Dieu of the place. Draughty rooms, impossible firegrates, indifferent sanitary arrangements, and general old-time appointments, make up the sum total of charity for the indigent sick without the walls of the castle of the Percys, whose present owner enjoys an income of four hundred thousand pounds sterling yearly.

There are only two wards in this hospital, three beds in one and two in the other. The smaller room is for women. There is one rather startlingly new piece of furniture in the operating room of which the Matron-nurse is justly proud. It consists of a combination chair, which on touching the proverbial button turns into operating-table, dentist chair, and sofa lounge as required. This novelty is placed in a chamber strictly in keeping with its own versatility, an operating room, theatre, bath, and lavatory in one. The matron herself is also in keeping with both furniture and room, for she is alternately matron, nurse, and sister. I came to the conclusion, on leaving, that the little nurse with sympathetic face and her collie pet dog by her side were the only bright touches in that rather dreary and uncomfortable infirmary. The cottage hospital is in the opposite direction from the infirmary and some short distance outside the town. It is now unoccupied. The building is comparatively new and consists of two large wards devoted to contagious diseases.

Alnwick is between two large centres Newcastle and Edinburgh, and the most difficult and interesting surgical cases that occur are generally forwarded to the hospitals of these towns.

